# Effect of *Phyllanthus amarus* Extract on 5-Fluorouracil-Induced Perturbations in Ribonucleotide and Deoxyribonucleotide Pools in HepG2 Cell Line

**DOI:** 10.3390/molecules21091254

**Published:** 2016-09-20

**Authors:** Jian-Ru Guo, Qian-Qian Chen, Christopher Wai-Kei Lam, Cai-Yun Wang, Feng-Guo Xu, Bu-Ming Liu, Wei Zhang

**Affiliations:** 1 State Key Laboratory of Quality Research in Chinese Medicines, Macau Institute for Applied Research in Medicine and Health, Macau University of Science and Technology, Taipa, Macau SAR, China; guojianrumust@gmail.com (J.-R.G.); qqchen@must.edu.mo (Q.-Q.C.); wklam@must.edu.mo (C.W.-K.L.); cywang@must.edu.mo (C.-Y.W.); 2 Key Laboratory of Drug Quality Control and Pharmacovigilance (Ministry of Education), China Pharmaceutical University, Nanjing 210009, China; fengguoxu@cpu.edu.cn; 3 Guangxi Key Laboratory of Traditional Chinese Medicine Quality Standards, Guangxi Institute of Traditional Medical and Pharmaceutical Sciences, Nanning 530022, China

**Keywords:** *Phyllanthus amarus* extract, 5-fluorouracil, ribonucleotides, deoxyribonucleotides

## Abstract

The aim of this study was to investigate the antitumor activities of *Phyllanthus amarus* (PHA) and its potential of herb–drug interactions with 5-Fluorouracil (5-FU). Cell viability, ribonucleotides (RNs) and deoxyribonucleotides (dRNs) levels, cell cycle distribution, and expression of thymidylate synthase (TS) and ribonucleotide reductase (RR) proteins were measured with 3-(4,5-dimethylthiazol-2-yl)-2,5-diphenyltetrazolium bromide (MTT) assay, high performance liquid chromatography tandem mass spectrometry (HPLC/MS/MS) method, flow cytometry and Western blot analysis, respectively. Our standardized PHA extract showed toxicity to HepG2 cells at high concentrations after 72 h exposure and induced G2/M cell cycle arrest. Combined use of 5-FU with PHA resulted in significant decreases in ATP, CTP, GTP, UTP and dTTP levels, while AMP, CMP, GMP and dUMP levels increased significantly compared with use of 5-FU alone. Further, PHA could increase the role of cell cycle arrest at S phase induced by 5-FU. Although PHA alone had no direct impact on TS and RR, PHA could change the levels of RNs and dRNs when combined with 5-FU. This may be due to cell cycle arrest or regulation of key enzyme steps in intracellular RNs and dRNs metabolism.

## 1. Introduction

Nucleoside analogs (NAs) have been developed for almost 50 years for treating cancers or viral infections [1]. NAs usually produce their major therapeutic effects through inhibition of DNA and RNA synthesis, regulation of key intracellular enzymes, and interference with cell signaling and intracellular metabolisms. These action mechanisms inevitably affect the natural ribonucleotides (RNs) and deoxyribonucleotides (dRNs) pool sizes, which play important roles in broad cellular functions such as DNA replication, proliferation and cell cycling [2,3]. Strategies to optimize intracellular RNs and dRNs pool sizes provide opportunities to potentiate antitumor effects of NAs and enhance cancer-cell selectivity, which have been proven beneficial in clinical trials [4,5].

5-Fluorouracil (5-FU) is a fluoropyrimidine anticancer agent that has been widely used to treat many types of cancers such as colorectal, liver and breast cancers [6,7,8]. Since its active metabolite, 5-fluoro-2′-deoxyuridine-5′-monophosphate (FdUMP), is an irreversible inhibitor of thymidylate synthase (TS), 5-FU incubation could lead to decrease of intracellular deoxythymidine triphosphate (dTTP) and increase of deoxyuridine monophosphate (dUMP) [9,10]. Meanwhile, other intracellular RNs and dRNs pool sizes were also perturbed by 5-FU due to complex metabolic networks of RNs and dRNs. In turn, the perturbation of RNs and dRNs could affect the action of 5-FU and its activity. 

Recently, there has been increasing interest worldwide in using herbal medicine combined with conventional chemotherapeutic agents for improving chemotherapeutic activity [11]. Many herbal medicines that can act as adjuvants for cancer therapeutics were found to enhance the efficacy of anti-cancer drugs as well as reducing the side effects and chemo-resistances [12]. *Phyllanthus amarus* (PHA) is a Chinese herbal medicine that has been used to treat various diseases including jaundice, dysentery, disorders, scabies and wounds [13]. There are various pathogens, toxins and risk factors leading to hepatocellular carcinoma, including hepatitis B virus (HBV), hepatitis C virus (HCV), alcohol, aflatoxin-B, diabetes, obesity, and non-alcoholic fatty liver disorders (NAFLD) [14,15,16,17]. PHA has a potential for treating hepatocellular carcinoma because it has been found to inhibit DNA polymerase of HBV and related hepatitis viruses and down regulate HBV mRNA transcription and translation [18,19,20]. In actual practice, cancer patients may receive PHA as adjuvant therapy intentionally or unintentionally, although the authorized information on its activity is not plentiful in the literature. Extracts of PHA could inhibit cytochromes P450 (CYP) CYP1A2, CYP2D6, CYP2E1 and CYP3A4 in a dose-dependent manner and thus raise the potential of herb–drug interactions. However, enzymes from the CYP family usually are not involved in metabolism of NAs [21]. Meanwhile, TS represents a critical target in cancer chemotherapy. Thus, it is critical to learn the possible pharmacokinetic interactions between PHA and 5-FU or other NAs since NAs are clinically important anticancer, anti-HBV and anti-HIV agents.

In this study, the cytotoxic effect of a chemically standardized PHA extract and the effect of this extract on 5-FU-induced perturbations in RNs and dRNs pool sizes were investigated in human hepatocellular carcinoma (HepG2) cells. Our results showed that high dosage and long treatment duration of PHA extract exerted a cytostatic effect on HepG2 cells. Meanwhile, low dosage of PHA also has a slightly synergistic effect and perturbation on RNs and dRNs pool sizes when combined with 5-FU.

## 2. Results and Discussion

### 2.1. Standarization of PHA Extract

PHA mainly contains lignans, triterpenes, alkaloids, and tannins and other components such as flavonoids, lactones and steroids [14]. Although Corilagin has been used as a marker compound, it seems to be insufficient to assess the quality of *Phyllanthus* [22]. We have developed An HPLC/TOFMS method together with chemometric methods has been developed for the quality assessment of *Phyllanthus* collected from different species and geographical locations [23]. A total of 59 compounds were identified and tentatively assigned structures from PHA extract by previously published method. By comparing the retention time and MS data with those of reference compounds, the contents of protocatechuic acid, corilagin, caffeic acid, ellagic acid, rutin, quercetin, luteolin, kaempferol, phyllanthin, niranthin, oleanolic acid, palmitic acid in the PHA were 3.91, 295.2, 15.2, 574.2, 217.7, 92.3, 0.10, 0.20, 792.3, 260.98, 16.3 and 12.9 µg/g, respectively.

### 2.2. Effect of PHA Extract on Growth of HepG2 Cells

**Figure 1:**
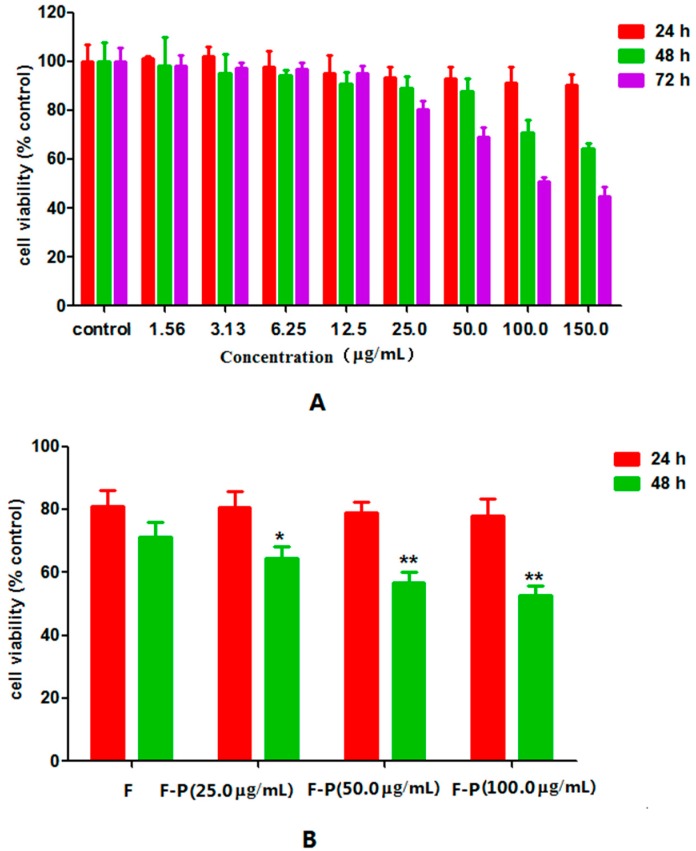
MTT results: (**A**) Cell viability of HepG2 cancer cells treated with different concentration of PHA for 24, 48 and 72 h; and (**B**) cell viability of combination treatment of 5-FU and PHA (* *p* < 0.05, ** *p* < 0.01, versus the 5-FU group).

In order to study the inhibition of PHA extract on cell growth, HepG2 cells were exposed to PHA extract at various concentrations (1.56, 3.13, 6.25, 12.5, 25.0, 50.0, 100.0 and 150.0 µg/mL) over different incubation periods (24, 48 and 72 h). High dosage and longtime treatment with PHA extract could inhibit proliferation of HepG2 (Figure 1A). The IC_50_ value of PHA extract on inhibition of HepG2 cells growth at 72 h was 114.0 ± 24.6 µg/mL. Furthermore, at low cytotoxicity dosage (50.0 µg/mL), PHA extract could also enhance cytotoxic activity of 5-FU but not significantly. Namely, exposure to 5-FU (50.0 µM) combined with 50.0 µg/mL PHA extract for 48 h decreased cell viability of HepG2 cells from 71.26% ± 3.93% to 54.66% ± 2.56% (Figure 1B, *p* < 0.01). At the same time, the value of combination index (CI) was 0.66 (Table 1). This suggests that there was a slightly synergy between these two drugs according to the data of cell viability and the value of CI. 

### 2.3. Multivariate Statistical Analysis

**Figure 2:**
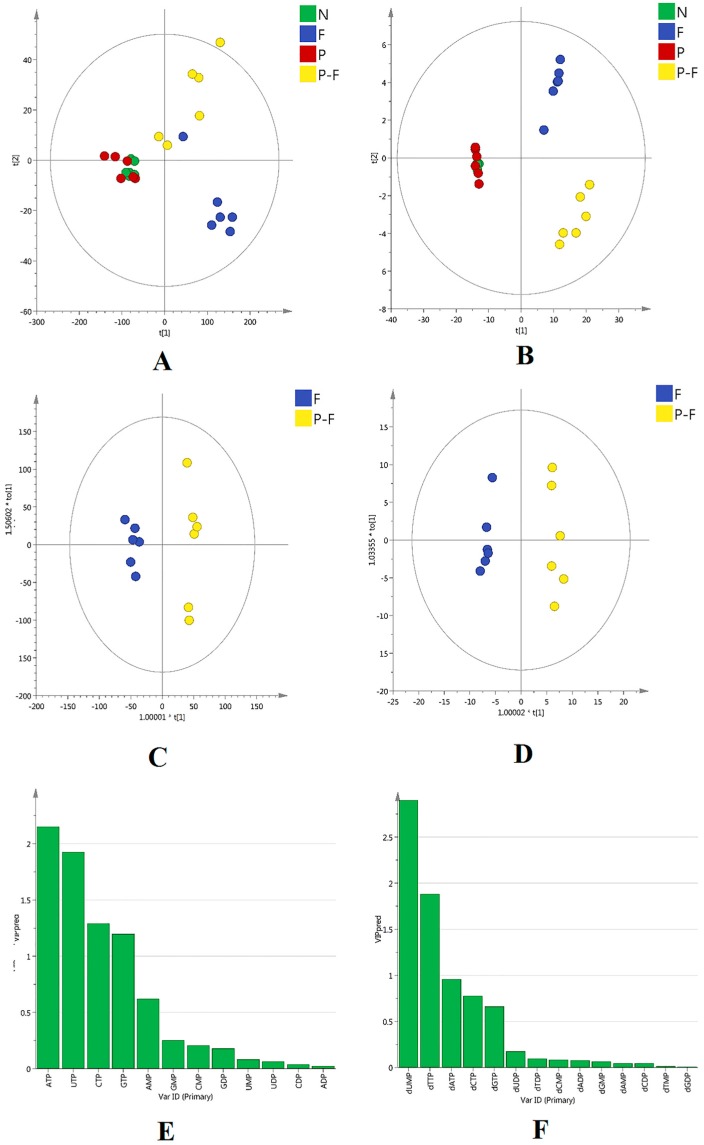
Score plots of PCA models comprising: (**A**) (RNs pool sizes, R_2_X = 0.998, Q^2^ = 0.993); and (**B**) (dRNs pool sizes, R_2_X = 0.978, Q^2^ = 0.915). Score plots of OPLS-DA models comprising: (**C**) (RNs pool sizes, R_2_X = 0.995, R_2_Y = 0.984, Q^2^ = 0.954); and (**D**) (dRNs pool sizes, R_2_X = 0.987, R_2_Y = 0.985, Q^2^ = 0.965). VIP plots of: (**E**) RNs pool sizes; and (**F**) dRNs pool sizes (N: Normal control group; F: 5-FU-treated group; P: PHA-treated group; P-F: combination group).

In order to understand and visualize the grouping trends, unsupervised principal component analysis (PCA) and supervised orthogonal partial least squares discriminant analysis (OPLS-DA) were performed using SIMCA-P version 14.0 software (Umetrics, Sweden). As shown in Figure 2A,B, clear group separation of sample points suggested that exposure to 5-FU with or without PHA extract made a significant difference in the profiles of RNs and dRNs pools, although PHA extract alone has no impact on intracellular RNs and dRNs pool sizes. Further, it can be seen from the OPLS-DA model in Figure 2C,D that F group and P-F group seemed easily classified into two groups with satisfactory discriminating ability, which suggested that PHA was able to stimulate the perturbation of RNs and dRNs levels induced by 5-FU. Finally, six RNs and dRNs including adenosine-triphosphate (ATP), cytidine triphosphate (CTP), guanosine triphosphate (GTP), uridine triphosphate (UTP), dUMP and dTTP were screened out using VIP > 1.0 and *p* < 0.05 (Figure 2E,F).

### 2.4. Effect of PHA Extract Incubation on RNs and dRNs Pool Sizes

**Figure 3:**
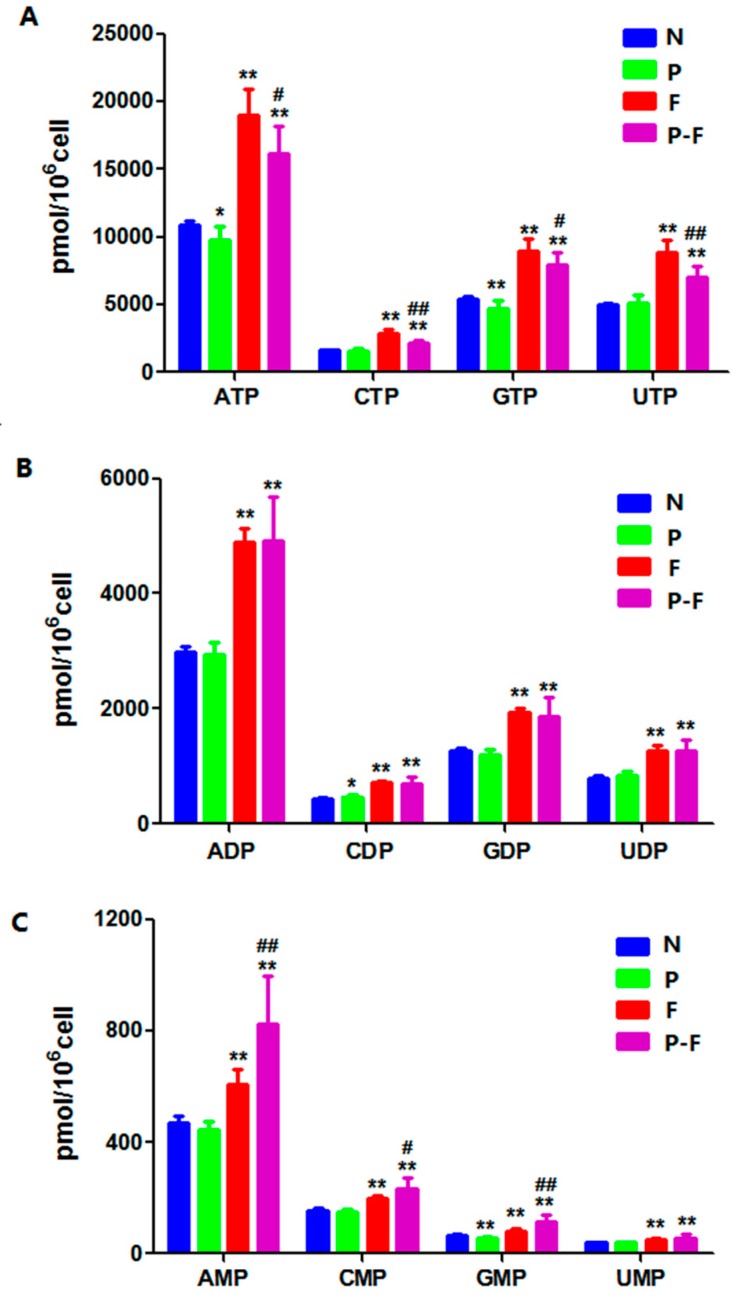
Levels of ribonucleotides in HepG2 cells after treatment with drugs for 24 h: (**A**) nucleoside triphosphate levels in every group; (**B**) nucleoside diphosphate levels in every group; and (**C**) nucleoside monophosphate levels in every group. Each group of data are represented as mean ± SD of three independent experiments. * *p* < 0.05 and ** *p* < 0.01, compared with the control group; ^#^ *p* < 0.05 and ^##^ *p* < 0.01, compared with the 5-FU group.

When HepG2 cells were exposed to the PHA extract alone, there were no remarkably changes in the levels of the RNs, except for the slight decrease in ATP, GTP, guanosine monophosphate (GMP) and the little increase in cytidine diphosphate (CDP). The effects of 5-FU on RNs and dRNs pool sizes in cells upon exposure to 5-FU for different durations have already been reported in our previous paper [24]. The levels of four nucleoside triphosphates exhibited similar increases after treatment of 5-FU with or without PHA extract (Figure 3 and Table 2). The effects of 5-FU on ribonucleotide triphosphates were possibly related to its cytostatic effect. However, it was interesting that ATP, CTP, GTP and UTP levels in the combination group significantly decreased compared with the 5-FU alone group. Meanwhile, there were significantly increases in adenosine monophosphate (AMP), cytidine monophosphate (CMP) and GMP levels after combined use with PHA extract. Nucleoside monophosphate kinase (NMPK) can phosphorylate monophosphate metabolites into diphosphates, which could be transformed to triphosphates metabolites by nucleoside diphosphate kinase (NDPK) [3]. Given that there was no effect of PHA extract on the level of nucleoside diphosphates, the nucleoside monophosphates and nucleoside triphosphates could have been affected by PHA. It was revealed that NMPK and NDPK may be the target for PHA, but this needs to be further studied.

**Figure 4:**
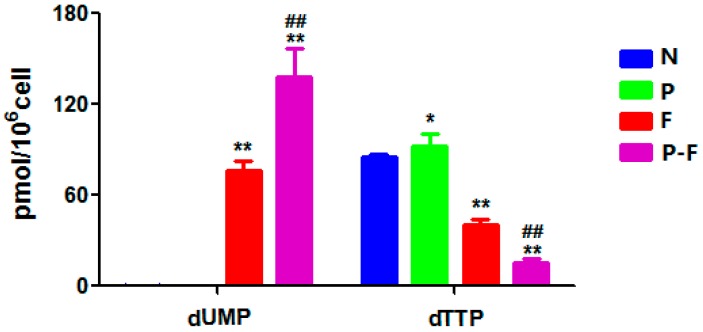
Levels of dUMP and dTTP after PHA and 5-FU treatments. Each group of data are represented as mean ± SD of three independent experiments. * *p* < 0.05 and ** *p* < 0.01, compared with the control group; ^##^ *p* < 0.01, compared with the 5-FU group.

**Figure 5:**
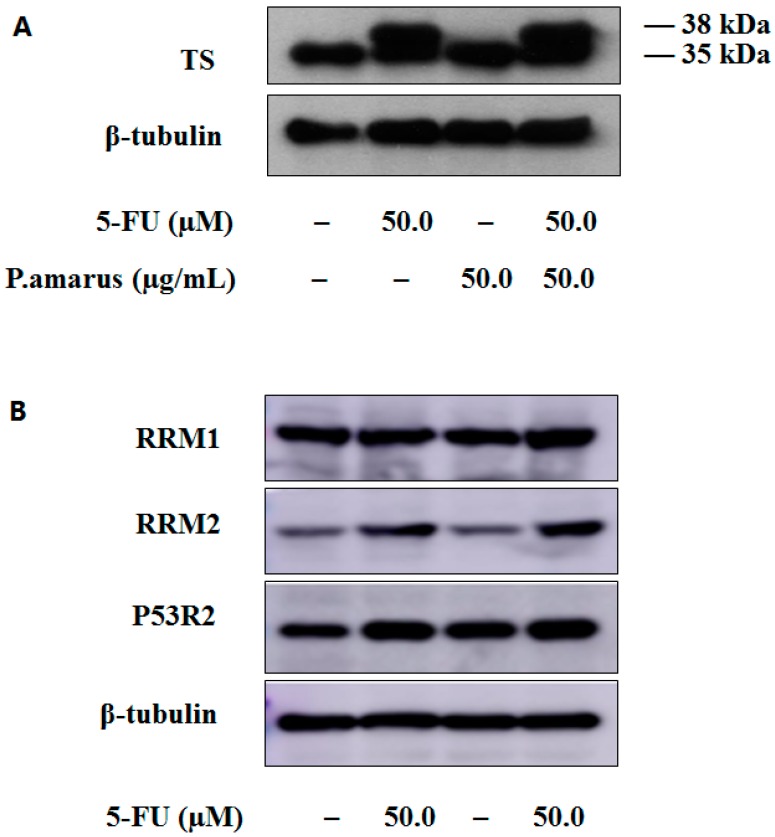
Western blot analysis in HepG2 cells: (**A**) expression of TS; and (**B**) expression of ribonucleotide reductase subunits M1 (RRM1) and M2 (RRM2), P53-controlled ribonucleotide reductase (P53R2).

Table 3 summarizes the levels of dRNs in each group. As far as we know, the accumulation of dUMP pools and the depletion of dTTP pools is the key mechanism of action of 5-FU via the inhibition of thymidylate synthase (TS) [25]. Furthermore, the level of dUMP in the 5-FU combined with PHA group was significantly higher than that of 5-FU group, while the dTTP pools reduced significantly (Figure 4). These findings suggested that PHA extract may enhance the effect of 5-FU through regulation of RNs and dRNs pool sizes. TS protein level was evaluated by Western blotting of HepG2 cell line. HepG2 cells were incubated for 24 h with 50.0 µM 5-FU alone, 50.0 µg/mL PHA alone and the combination of these two drugs, respectively. As shown in Figure 5A, the level of TS protein remained unchanged after exposure to PHA extract. Expectedly, treatment with 5-FU induced the formation of two distinct bands of TS, one at 35 kDa and the other one at 38 kDa approximately. The lane at 35 kDa represents the normal TS. The second bands of TS with higher molecular mass represent the conjugation, which is formed by FdUMP, TS and CH2-THF (5, 10-methylene-tetrahydrofolate) [26]. However, there was no inhibitory effect on this enzyme after combined use with PHA extract. Since deoxycytidine triphosphate (dCMP) is a precursor of dUMP [27], the increase of dUMP in combination group could be caused by increase of dCMP. These possibilities are under investigation.

### 2.5. PHA extract Induced Cell Cycle Arrest in HepG2 Cells

**Figure 6:**
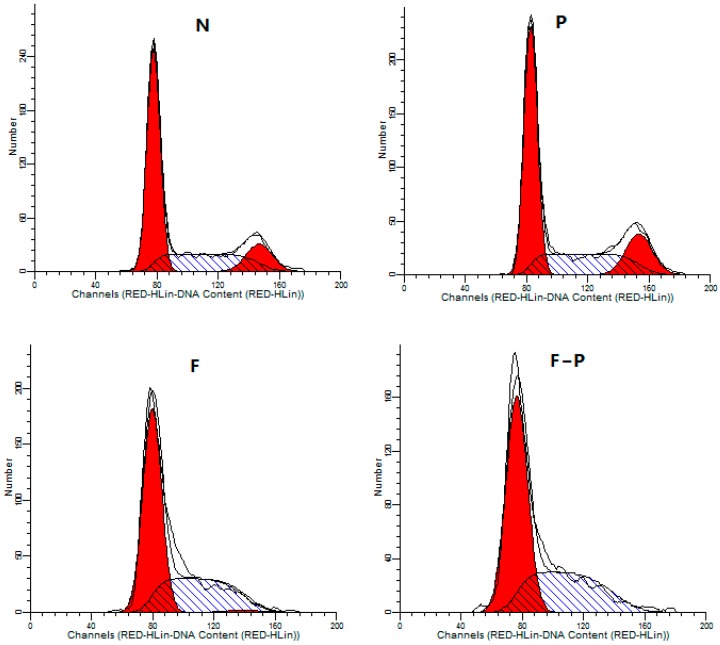
Cell cycle effects of 5-FU and PHA in HepG2 cells after 24 h exposure.

After exposure to 5-FU (50.0 µM), PHA(50.0 µg/mL) or combination for 24 h, cell cycle arrest in HepG2 cells was investigated using flow cytometry. Results showed that treatment for 24 h with PHA extract alone induced the cells arrest in G2/M phase slightly. However, there were no significant differences in other phases (Figure 6). At the same time, 5-FU group showed a remarkable inhibition in the G0/G1 and S phases by a decreased distribution in the G2/M phase. Compared with 5-FU group, combined treatment with 5-FU and PHA extract induced a higher proportion arrest in S phase and decreased distribution in G0/G1 phase in HepG2 cells. This suggested that PHA extract could increase the role of cell cycle arrest at S phase induced by 5-FU in HepG2 cells.

Human ribonucleotide reductase (RR) is composed of three known subunits, RRM1 (large subunit), RRM2 (small subunit), and encoded P53-controlled ribonucleotide reductase (P53R2), which are differentially regulated during the cell cycle. R1 protein concentration is relatively constant throughout the cell cycle. M2 protein is low outside S phase [28,29]. Compared with the control group, the expression of RRM2 was increased after 5-FU treatment with or without PHA extract, which was caused by cell cycle arrest at S phase (Figure 5B). Thus high levels of deoxyadenosine diphosphate (dADP), deoxycytidine diphosphate (dCDP), deoxyadenosine triphosphate (dATP) and deoxycytidine triphosphate (dCTP) were associated with high level of RRM2. As already noted, dTTP stimulates the formation of deoxyguanosine diphosphate (dGDP) and hence of deoxyguanosine triphosphate (dGTP). The subsequent decrease in the levels of dGDP and dGTP in F and P-F groups may be, at least in part, caused by decrease of dTTP due to inhibition of 5-FU on TS.

## 3. Materials and Methods

### 3.1. Materials

LC-MS grade methanol, acetonitrile and acetic acid were purchased from Anaqua Chemical Supply Co., Houston, TX, USA. Hexylamine (HA), diethylamine (DEA), trioctylamine, 1,1,2-trichlorotrifluoroethane, stable isotope labeled adenosine-^13^C_10_^15^N_5_-triphosphate (ATP^13^C^15^N), dimethyl sulfoxide (DMSO), trypsin-EDTA solution and 3-[(4,5)-dimethylthiazol-2-yl]-2,5-diphenyl tetrazolium bromide (MTT) were purchased from Sigma Aldrich Chemical Co., St. Louis, MO, USA. Ultra-pure water was obtained from a Milli-Q Gradient Water System (Millipore Corp., Bedford, MA, USA). For culturing cells, phosphate buffered saline, pH = 7.8 (PBS), Dulbecco’s Modified Eagle Medium (DMEM), penicillin–streptomycin solution and fetal bovine serum (FBS) were obtained from Gibco Invitrogen Corp., Carlsbad, CA, USA. Human hepatocellular cancer HepG2 cell line was supplied by American Type Culture Collection (ATCC), Rockville, MD, USA.

### 3.2. Preparation of PHA Extract

PHA was collected from the Hepu town of Beihai, Guangxi province, China and identified by Professor Buming Liu at the Guangxi Key Laboratory of Traditional Chinese Medicine Quality Standards, China. The dried powder of PHA (about 5 g) was accurately weighed into a 250 mL flask and immersed in 100 mL of 75% ethanol (*v*/*v*) overnight. The mixture was ultrasonically extracted at room temperature for 1 h before the extract was filtered. The filtrate was condensed by a rotary evaporator followed with vacuum drying at 60 °C. A total of 59 compounds were identified and tentatively assigned structures for the establishment of fingerprint by previously published method [23]. By comparing the retention time and MS data with those of reference compounds, the contents of protocatechuic acid , corilagin, caffeic acid, ellagic acid, rutin, quercetin, luteolin, qaempferol, phyllanthin, niranthin, oleanolic acid, palmitic acid in the PHA were 3.91, 295.2, 15.2, 574.2, 217.7 ,92.3, 0.10, 0.20, 792.3, 260.98, 16.3 and 12.9 µg/g, respectively.

### 3.3. LC/MS/MS Assay

This was performed on a Thermo Fisher TSQ LC-MS/MS system consisted of an Accela Autosampler, an Accela pump and a Quantum Access triple quadrupole mass spectrometer (Thermo Fisher Scientific Co., San Jose, CA, USA). Data acquisition was performed with the Xcalibur software version 2.0.7, and data processing using the Thermo LCquan 2.5.6 data analysis program (Thermo Fisher). The chromatographic separation was achieved using an XTerra-MS C18 column (150 mm × 2.1 mm i.d., 3.5 µm, Waters Corp., Milford, MA, USA). The two eluents were: (A) 5 mM HA–0.5% DEA in water, pH adjusted to 10 with acetic acid; and (B) 50% acetonitrile in water. The mobile phase consisted of a linear gradient of A and B: 0–15 min, 100%–80% A (*v*/*v*); 15–35 min, 80%–70% A; 35–45 min, 70%–45% A; 45–46 min, 45%–0% A; 46–50 min, 0%–0% A; 50–51 min, 0%–100% A; 51–70 min, 100–100 % A. The liquid flow-rate was set at 0.3 mL/min, and the column temperature was maintained at 35 °C. For all dRNs, the following optimized parameters were obtained. The sheath gas pressure reached 40 psi. The ion spray voltage was set at 3000 V for negative mode and 4000 V for positive mode at a temperature of 350 °C and auxiliary gas pressure of 15 psi. Quantification was performed using multiple reactions monitoring (MRM) as previously published [24].

### 3.4. Cell Culture

HepG2 cells were cultured in DMEM medium supplemented with 10% fetal bovine serum (FBS), 100 units/mL penicillin, 100 µg/mL streptomycin in a 37 °C humidified incubator with a 5% CO_2_ atmosphere. HepG2 cells were seeded in 100 mm by 20 mm dishes (Corning Inc., Corning, NY, USA). After overnight culture, cells were divided into different groups as follows: control group (N), cells was incubated with medium alone; 5-FU group (F), cells were exposed to 50.0 µM of 5-FU for 24 h; PHA group (P), cells were exposed to 50.0 µg/mL of PHA extraction for 24 h; and combination group (P-F), cells were exposed to 50.0 µM of 5-FU combined with 50.0 µg/mL of PHA extraction for 24 h. An extra dish of cells was incubated for cell counting on the day of cell harvest for normalization of nucleotide pools, and the viability assessed by tryphan blue exclusion assay.

### 3.5. Preparation of Cell Pellets

Monolayer HepG2 cells were washed with ice-cold PBS once and were trypsinized with 0.25% trypsin-EDTA. Cells from two or three dishes were then re-suspended with 12 mL ice-cold PBS. After centrifugation at 1000 rpm for 5 min, the cell pellet was washed with 1 mL ice-cold PBS again and spun down at 1000 rpm for 5 min. The cell pellet was incubated with 150 µL of 15% trichloroacetic acid (TCA) containing 7.5 µL of 20.0 µM ATP^13^C^15^N as internal standard and placed on ice for 10 min. After centrifugation at 13,500 rpm for 15 min in the cold room, the acidic supernatant was separated and neutralized twice with 80 µL mixture of trioctylamine and 1,1,2-trichlorotrifluoroethane (45:55 *v*/*v*). Samples were stored at −80 °C until analysis within two days.

### 3.6. MTT Assay

The inhibitory effect of different groups on HepG2 cells was determined by the cytotoxic MTT assay. HepG2 cells were seeded in 96 wells plate (LabServ, Thermo Fisher Scientific Co., Beijing, China) at 1 × 10^4^ cells/well. After incubation, they were treated with indicated concentrations of 5-FU, PHA or combination for 24, 48 and 72 h. MTT solution (final concentration of 0.5 mg/mL in medium) was added to each well and incubated further for 4 h. The medium was removed, and 100 μL of DMSO was added to each well to dissolve the purple crystals of formazan. Absorbance was measured at 570 nm with a microplate UV/VIS spectrophotometer (Infinite M200 PRO, Tecan Austria GmbH 5082, Grödig, Austria); reference wavelength was 650 nm. The cell number was determined using a hemocytometer. IC_50_ (half maximal (50%) inhibitory concentration) values of 5-FU were calculated by GraphPad Prism software (GraphPad Software, Inc., San Diego, CA, USA). Cell viability (%) = (OD (treated) − OD (blank))/(OD (control, untreated) − OD (blank)) × 100. Drug interaction was evaluated by the combination index (CI) methods, whereas CI < 1, CI = 1, or CI > 1 indicates synergism, additive effect or antagonism, respectively [30,31]. CI values were calculated by CompuSyn software (ComboSyn, Inc., Paramus, NJ, USA).

### 3.7. Cell Cycle Analysis

The cell cycle analysis was determined as previously described [32]. In brief, cells were seeded at 4.5 × 10^5^ cells/well in 6-well culture plates in duplicate, and incubated with indicated concentrations of 5-FU, PHA extract or combination for 24 h. They were then harvested and fixed in 70% (*v*/*v*) cold ethanol overnight at 4 °C. The fixed cells were collected by centrifugation and re-suspended in PBS and incubated with 5 mg/mL propidium iodide (Sigma-Aldrich Co.) and 10 mg/mL RNase A (Sigma-Aldrich) at room temperature for 30 min in the dark. The cells were then analyzed on a flow cytometer (Muse™ cell analyzer, Merck Millipore, Darmstadt, Germany). Finally, the percentages of cells in different phases (G0/G1, S and G2/M) were calculated using Modfit software (Verity Software House, Verity Software House, Topsham, ME, USA).

### 3.8. Western Blot Analysis

The cells were treated with 5-FU, PHA extract and the combination of them for 24 h, respectively. At the end of the incubation time, every group cells were harvested and lysed in RIPA buffer (Cell Signaling Technologies Inc., Beverly, MA, USA). Bradford reagent (Bio-Rad Laboratory, Hercules, CA, USA) was used to determine protein concentration. Then, the cell lysates were mixed with 5 × SDS-loading buffer (4:1, *v*/*v*) and heated at 100 °C with locked capping for 5 min. The cell lysates (40 μg) were subjected to 10% SDS-PAGE. After electrophoresis, the cell extracts from SDS-PAGE were transferred to nitrocellulose membrane (Millipore Co.). Then, the membranes were incubated with rabbit RRM1 (D12F12) antibody (Dilution ratio = 1:1000; Cell Signaling Technology, Inc.), RRM2 [EPR11820] antibody (Dilution ratio = 1:1000; abcam^®^, Cambridge, UK), p53R2 [EPR8816] antibody (Dilution ratio = 1:1000; abcam^®^), thymidylate synthase (D5B3) antibody (Dilution ratio = 1:1000; Cell Signaling Technology, Inc.) and β-tubulin antibody (Dilution ratio = 1:2000; Santa Cruz Biotechnology, CA, USA) overnight at 4 °C. Furthermore, the membranes were incubated with HRP-conjugated antibodies (Dilution ratio = 1:5000; Cell Signaling Technology, Inc.) for one hour. Visualization of the protein bands by using the enhanced chemiluminescence reagents (Invitrogen, Paisley, Scotland, UK). The bands analyzed by using the Image J 1.46r software (National Institutes of Health, Bethesda, MD, USA).

## 4. Conclusions

In this study, the interaction of combined use of PHA and 5-FU (50.0 µM) on cell growth and cell cycle progression were investigated. Although PHA alone has no significant impact on the levels of RNs and dRNs, combined use of 5-FU with PHA enhanced cytotoxic activity through regulation of ribonucleotides and deoxyribonucleotides pool sizes which might be due to the cell cycle arrest or regulation of key enzyme steps in intracellular RNs and dRNs metabolism. Combined pharmacotherapy is a popular strategy that would enhance the anti-cancer activities of cytotoxic drugs while reducing cancer cells’ chemo-resistance. The use of PHA for the treatment of liver diseases has a long history in several countries. TS and RR represent two critical targets in cancer chemotherapy. Although PHA has no direct effect on TS and RR, it could perturb the levels of RNs and dRNs and could induce cell cycle arrest when combined with 5-FU. Thus, interaction between PHA and 5-FU could occur and may lead to serious side effect because imbalances in the four dNTPs pools have genotoxic consequences.

## Figures and Tables

**Table 1:** The combination index (CI) values of combination treatment of 5-FU and PHA in HepG2 cells.

Time (h)	PHA (µg/mL)		
	25.0	50.0	100.0
24	1.16	1.23	1.37
48	0.76	0.66	0.71

**Table 2:** Levels of RNs in HepG2 cells of every group (pmol/10^6^ cell).

	MRM Transition	Control	F	P	P-F
ATP	506.0→159.0	10904.7 ± 275.1	18975.6 ± 1728.7 **	9775.0 ± 936.9 *	16173.0 ± 1784.1 **^,#^
ADP	426.0→328.0	2985.5 ± 89.5	49033 ± 207.5 **	2933.1 ± 190.5	4916.2 ± 709.2 **
AMP	346.0→79.3	469.5 ± 21.1	608.5 ± 49.7 **	444.1 ± 27.6	823.9 ± 156.21 **^,##^
CTP	482.0→384.0	1601.3 ± 41.7	2860.0 ± 269.9 **	1523.9 ± 162.1	2116.5 ± 213.2 **^,##^
CDP	402.0→159.0	430.3 ± 15.9	707.9 ± 35.9 **	468.4 ± 28.9 *	697.7 ± 98.7 **
CMP	322.0→79.4	153.4 ± 7.01	200.2 ± 7.2 **	148.3 ± 8.6	232.2 ± 37.0 **^,#^
GTP	522.0→423.9	5428.2 ± 110.7	8923.2 ± 807.2 **	4668.0 ± 547.8 **	7912.1 ± 807.8 **^,#^
GDP	442.0→344.0	1263.4 ± 45.3	1935.3 ± 64.0 **	1203.3 ± 87.2	1858.9 ± 305.0 **
GMP	362.0→79.30	67.0 ± 2.8	81.3 ± 8.2 **	55.0 ± 4.1 **	115.8 ± 22.5 **^,##^
UTP	482.9→385.0	4989.0 ± 114.2	8858.3 ± 774.9 **	5097.9 ± 517.1	7045.6 ± 741.2 **^,##^
UDP	403.0→159.0	792.9 ± 38.2	1280.0 ± 69.9 **	844.9 ± 55.7	1261.1 ± 189.2 **
UMP	323.0→79.3	39.3 ± 1.8	49.0 ± 4.2 **	38.6 ± 2.3	56.4 ± 12.4 **

Note: All data are expressed as mean ± SD values by three independent experiments. *P* values of less than 0.05 (* *p* < 0.05, ** *p* < 0.01, versus the control group; ^#^ *p* <0.05, ^##^ *p* < 0.01, compared with the 5-FU group) are considered significant.

**Table 3:** Levels of dRNs in HepG2 cells of every group (pmol/10^6^ cell).

	MRM Transition	Control	F	P	P-F
dATP	490.0→159.1	30.34 ± 0.5	113.11 ± 10.2 **	32.45 ± 1.8 *	126.9 ± 16.4 **
dADP	410.0→79.0	0.0568 ± 0.005	0.2789 ± 0.02 **	0.0526 ± 0.003	0.3414 ± 0.05 **^,#^
dAMP	330.0→79.0	0.0068 ± 0.002	0.0354 ± 0.003 **	0.007 ± 0.001	0.0525 ± 0.01**^,##^
dCTP	465.9→159.0	28.48 ± 0.6	138.3 ± 12.3 **	24.00 ± 1.8 **	126.6 ± 18.8 **
dCDP	386.0→79.0	0.0297 ± 0.005	0.1185 ± 0.01 **	0.0189 ± 0.002 **	0.1446 ± 0.03 **
dCMP	306.0→79.0	0.0499 ± 0.006	0.0394 ± 0.007 *	0.0304 ± 0.008 **	0.0923 ± 0.016 **^,##^
dGTP	581.0→152.1	40.35 ± 2.3	12.81 ± 0.50 **	34.22 ± 3.4 **	9.52 ± 0.84 **^,##^
dGDP	501.0→152.0	2.12 ± 0.09	0.13 ± 0.07 **	2.22 ± 0.4	0.13 ± 0.06 **
dGMP	421.0→152.0	2.92 ± 0.1	0.28 ± 0.07 **	2.72 ± 0.4	0.35 ± 0.09 **
dUTP	467.0→159.0	*UDL	*UDL	*UDL	*UDL
dUDP	387.0→79.0	0.02 ± 0.01	0.22 ± 0.07 **	1.52 ± 3.4	0.59 ± 0.39 **^,#^
dUMP	307.0→79.0	0.17 ± 0.03	76.38 ± 5.7 **	0.15 ± 0.05	137.79 ± 17.3 **^,##^
dTTP	481.0→159.1	85.54 ± 1.0	40.61 ± 3.0 **	92.77 ± 7.2 *	15.73 ± 1.8 **^,##^
dTDP	401.0→79.0	2.66 ± 0.2	1.25 ± 0.05 **	3.11 ± 0.1 **	1.14 ± 0.07 **^,##^
dTMP	321.0→79.0	0.0435 ± 0.005	0.004 ± 0.0008 **	0.049 ± 0.003 *	0.0071 ± 0.004 **

Note: All data were expressed as mean ± SD values by three independent experiments. *P* value of less than 0.05 (* *p* < 0.05, ** *p* < 0.01, versus the control group; ^#^ *p* < 0.05, ^##^ *p* < 0.01, compared with the 5-FU group) are considered significant; *UDL, under detected limit of assay.
